# Peanut and hazelnut occurrence as allergens in foodstuffs with precautionary allergen labeling in Canada

**DOI:** 10.1038/s41538-021-00093-4

**Published:** 2021-05-11

**Authors:** Emilie Manny, Sébastien La Vieille, Virginie Barrere, Jérémie Théolier, Samuel Benrejeb Godefroy

**Affiliations:** 1 Food Risk Analysis and Regulatory Excellence Platform (PARERA), Institute of Nutrition and Functional Foods and Department of Food Science, Université Laval, Québec, Canada; 2 Food Directorate, Health Canada, 251 Sir Frederick Banting Driveway, Ottawa, ON Canada

**Keywords:** Agriculture, Immunochemistry

## Abstract

Precautionary allergen labeling (PAL) is widely used by food industries. Occurrence studies revealed that few analyzed products contained the allergen(s) present in the statement, but little is known in Canada. To improve manufacturing practices and better manage allergen cross-contamination, occurrence data is needed to determine the exposure of allergic individuals eating those products. Samples were analyzed for peanuts (*n* = 871) and hazelnuts (*n* = 863) using ELISA methods. Within samples analyzed for peanuts, 72% had a PAL (*n* = 628), 1% had peanuts as a minor ingredient (*n* = 9) and 27% were claimed “peanut-free” (*n* = 234). Most hazelnut samples had a PAL for tree nuts/hazelnuts (94%; *n* = 807) with 6% claimed “nut-free” (*n* = 56). Peanuts and hazelnuts were found in 4% (0.6–28.1 ppm) and 9% (0.4–2167 ppm) of all samples, respectively. Chocolates were mostly impacted; they should be treated apart from other foods and used in risk assessments scenarios to improve manufacturing practices, reducing unnecessary PAL use.

## Introduction

About 1.2 and 1.4% Canadians suffer from peanut and tree nut allergy respectively^1^, which means more than 400 000 Canadians are affected per allergen, according to the 2016 Census (https://www12.statcan.gc.ca/census-recensement/2016/dp-pd/hlt-fst/pd-pl/Comprehensive.cfm). Being food allergens, peanuts and tree nuts such as hazelnuts can trigger objective reactions and anaphylaxis^2,3^. According to the Canadian Regulations, peanuts and hazelnuts are considered as priority allergens in Canada and need to be clearly indicated in the ingredients’ list when added intentionally (https://laws-lois.justice.gc.ca/PDF/C.R.C.,_c._870.pdf). This allows allergic consumers to identify the food allergens they are allergic to more easily and avoid allergic reactions.

Peanut and hazelnuts are widely used in food products such as chocolate, cookies, snacks (e.g., nut mixes, nutrition bars) and baked goods (e.g., cakes, pastries)^4^. During processing of these foods, allergen cross-contamination may occur. Food industries can use precautionary allergen labeling (PAL) on a voluntary basis to notify their consumers that peanuts and hazelnuts can be present, even if not intentionally added^5^. However, PAL use was reported to lead to a decrease in the food offer towards allergic consumers^6,7^. Due to the high frequency of PAL use, some consumers have started adopting a risky behavior by eating products with PAL repeatedly after not experiencing objective reactions^5^. Indeed, combined past occurrence studies based on analytical methods like enzyme-linked immunosorbent assays (ELISA), or polymerase chain reaction (PCR), have shown about 10% of products contained the mentioned allergens (peanuts and/or hazelnuts) in the PAL statement when all data was pooled together, and consequently, 90% of the products with PAL did not contain any food allergen^6,8–15^.

To provide guidance to food industries on the use of PAL in processed foods, risk assessments using Canadian-based occurrence data must be undergone to develop allergen management tools such as allergen action levels. In 2014, an industrial consortium met to determine allergen action levels in Australia & New-Zealand: the VITAL (Voluntary Incidental Trace Allergen Labeling) program^16,17^. Food industries participating to the VITAL program need to determine their allergen trace levels and make sure they are below the threshold determined by the consortium if they want to remove PAL from the labeling (http://www.allergenbureau.net/downloads/vital/VSEP-Summary-Report-Oct-2011.pdf). VITAL action levels were recently updated with new reference doses (peanuts = 0.2 mg; hazelnuts = 0.1 mg)^18^. Otherwise, if the allergen trace levels are above the threshold, “may be present” must be indicated on the label, as the allergen is not added intentionally and is present because of inevitable cross-contamination^17^. Canadian allergen action levels (like VITAL thresholds) could be used by food industries in Canada to conduct risk assessments and help determine the necessary use of PAL. However, only one allergen occurrence study on Canadian-based data in foodstuffs with PAL was found in the literature for peanuts^19^. In this study, chocolate bars with a PAL for peanut did not contain peanuts when they were manufactured in North America, whereas 25% of the European bars with PAL did contain peanuts^19^, which highly suggested overuse of PAL.

In addition, few articles in other countries addressed the lot-to-lot variability^6,9,13,14^ and the differences between brands, industry size or origin^10,19^, and were mostly focusing on the PAL statement used^6,10,14^. Articles addressing lot-to-lot variability analyzed up to four different lots, but for few products only^9,13^. Some authors concluded that when a product contained the allergen mentioned in the PAL statement, it was more likely that it would always contain allergen traces^9^. The articles investigating differences between PAL statements (“may contain” vs. “may contain traces”) all concluded that there were no differences between the statements used^6,10,14^.

This paper addresses the data gap related to peanut and hazelnut occurrence in foodstuffs with PAL in Canada, to enable occurrence-based risk assessment strategies that will help determine allergen action levels to improve the use of PAL. Using a sample plan based on past occurrence studies and the Canadian Food Inspection Agency (CFIA) food recall database, products with a PAL for peanuts and/or hazelnuts were analyzed for peanut and/or hazelnut as unintended allergens using an ELISA method. The PAL statement used, lot-to-lot variability, origin and brand types were investigated in this article to address the gaps in allergen occurrence research in Canada and have a more representative portrait of cross-contamination in products with PAL for peanuts and hazelnuts. The occurrence data generated would support risk assessment scenarios based on Canadian data.

## Results

The summary of analyzed products for each food category and allergen are presented in Table 1 (products with PAL, or with the allergen present as a minor ingredient) and Table 2 (products bearing an allergen-free claim).

**Table 1:** Summary of purchased products per allergen and by food category with PAL or peanuts present as a minor ingredient.

Food category	Peanuts									Hazelnuts						
	Products	Lots (/5)					Samples (Total)	Samples with PAL/Total	Samples with peanuts^a^/Total	Products	Lots (/5)					Samples (Total)
		1	2	3	4	5					1	2	3	4	5	
Baked goods	32	32	16	10	9	7	74	74/74	0/74	48	48	31	22	15	13	129
Baking mixes	12	12	11	9	7	6	45	45/45	0/45	16	16	15	13	8	6	58
Candies	40	40	29	23	3	2	97	97/97	0/97	41	41	30	25	5	4	105
Cereal bars & products	31	31	29	25	12	10	107	101/107	6/107	35	35	31	27	10	9	112
Chocolate products	31	31	29	25	12	10	86	86/86	0/86	42	42	37	34	19	16	148
Cookies	30	30	24	18	6	5	99	99/99	0/99	30	30	26	18	16	13	103
Savoury biscuits	13	13	11	8	6	3	41	41/41	0/41	23	23	18	12	6	3	62
Snacks	34	34	27	22	4	1	88	85/88	3/88	35	35	28	23	3	1	90
Total	223	223	173	133	61	47	637	628/637	9/637	270	270	216	174	82	65	807

^a^Peanuts present as a minor ingredient (last ingredient of the ingredients’ list).

**Table 2:** Purchased products per allergen and food category with an allergen-free statement.

Food category	Peanuts							Hazelnuts						
	Products	Lots (/5)					Samples (Total)	Products	Lots (/5)					Samples (Total)
		1	2	3	4	5			1	2	3	4	5	
Baked goods	4	4	4	3	2	2	15	1	1	1	1	0	0	3
Baking mixes	0	0	0	0	0	0	0	0	0	0	0	0	0	0
Candies	6	6	6	5	5	1	23	2	2	2	2	2	1	9
Cereal bars & products	8	8	7	7	7	6	35	0	0	0	0	0	0	0
Chocolate products	9	9	7	7	7	7	37	4	4	3	3	3	2	15
Cookies	21	21	21	20	18	18	98	2	2	2	2	0	0	6
Savoury biscuits	9	9	9	8	0	0	26	7	7	7	6	3	0	23
Snacks	0	0	0	0	0	0	0	0	0	0	0	0	0	0
Total	57	57	54	50	39	34	234	16	16	14	14	8	4	56

### Peanut occurrence

A total of 871 samples were analyzed for peanuts in this study. PAL statements were present in 72% of samples (628/871) and 1% (9/871) were labeled as containing peanuts as a minor ingredient (Table 1). Samples with a peanut-free claim constituted 27% (234/871) of all samples (Table 2).

Thirty-eight (38) samples were positive for peanuts out of 871 (4%), of which 31 presented a PAL statement for peanuts (31/628; 5%), six had peanuts as a minor ingredient (6/9; 67%), and another one was presented as manufactured in a peanut-free facility (1/234; 0.4%). For all analyzed samples, the peanut protein concentration ranged from 0.6 to 28.1 ppm for samples with PAL, from 0.6 to 1.3 ppm for samples with peanuts as a minor ingredient, and 0.7 ppm for the sample with a peanut-free claim.

Regardless of the allergens listed in the PAL statement, “may contain” was mostly used (*n* = 800; 92%) followed by “may contain traces” (*n* = 19, 2%), “may contain ingredients” (*n* = 12; 1%), and other PAL statements such as “made in a facility that also processes” (*n* = 22; 3%). The remaining samples (*n* = 18; 2%) did not present a PAL statement at all (for any allergen) and did not contain peanuts as minor ingredients but presented a peanut-free claim only.

**Fig. 1:**
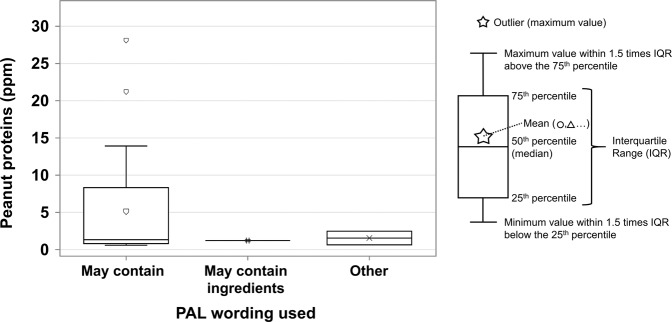
Peanut protein concentration by PAL statement used. Each box represents a different PAL wording and has a different color-symbol combination. The “May contain” category presents two outliers. There were no positive results for “May contain traces” in products with a PAL for peanuts.

Within the 31 positive samples with a PAL for peanuts, 28 (90%) had “may contain” and contained from 0.6 to 28.1 ppm peanut proteins, one had “may contain ingredients” and contained 1.2 ppm peanut proteins, and two had another type of PAL (“may contain proteins”) and contained 0.6 and 2.5 ppm peanut proteins, respectively. The differences between PAL statements can be observed in Fig. 1. No products with “may contain traces” did contain the allergen mentioned in that PAL.

**Fig. 2:**
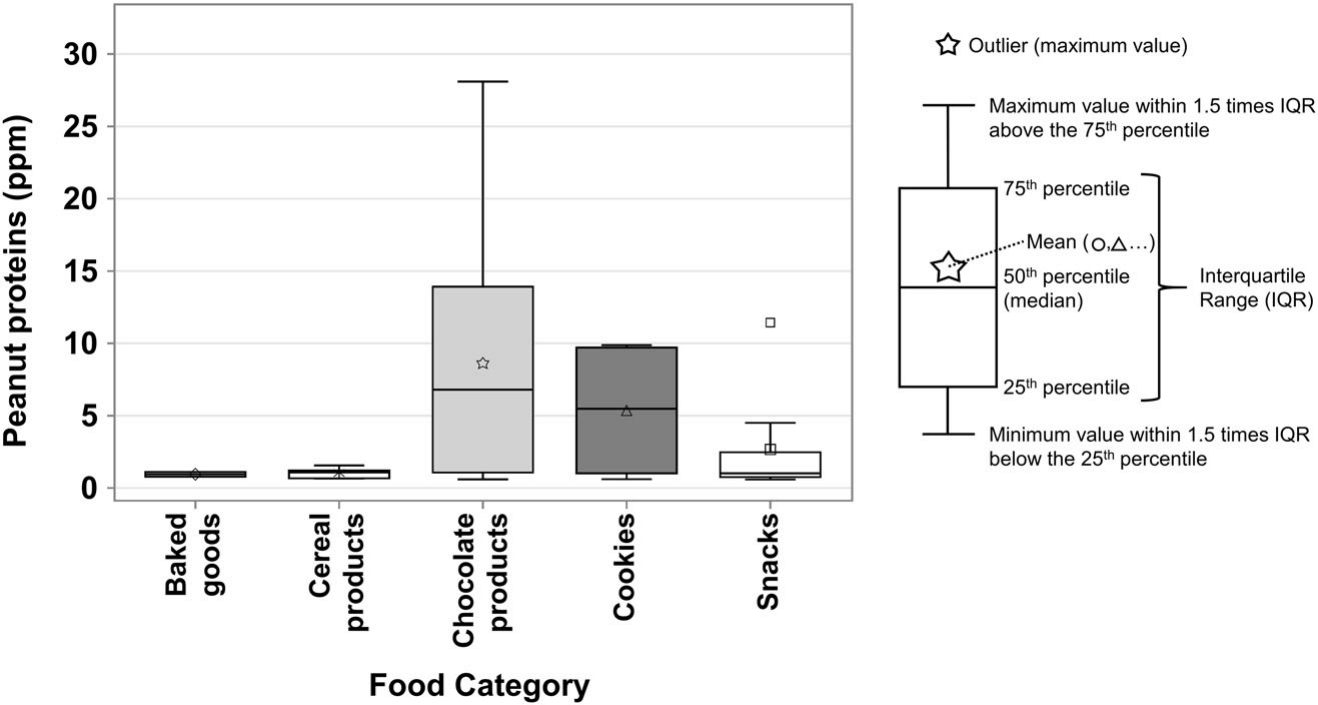
Peanut protein concentration by food category. Each box corresponds to a different food category and has a different color-symbol combination. The “Snacks” category presents one outlier. The other food categories (Baking mixes, Candies, Savoury biscuits) had no positive results for peanuts.

There were high variations between peanut protein concentration from positive samples with PAL of some food categories, as shown in Fig. 2. The mean peanut protein concentration and standard deviations for chocolate products (*n* = 11), cookies (*n* = 4), and snacks (*n* = 9) were respectively of 8.6 ± 9.3 ppm, 5.4 ± 5.0 ppm, and 2.7 ± 3.5 ppm (Fig. 2). For chocolate products and snacks, the standard deviation was above the mean (e.g., 8.6 < 9.3 for chocolate products). Baked goods (*n* = 2) and cereal products (*n* = 5) had lower protein concentrations and were showing less variations between different samples than the other categories. The occurrences of products with PAL of all food categories are 3% for baked goods (2/74), 5% for cereal products (5/101), 13% for chocolate (11/86), 4% for cookies (4/99), 11% for snacks (9/85), and 0% for remaining categories (baking mixes, candies, savoury biscuits). The occurrence of products bearing a peanut-free claim was 0% was all food categories, except cookies (1%; 1/98).

Twenty-three (23) products had contained peanuts in one or more lots. Only two products with a PAL for peanuts, one chocolate and one cookie, had all their 3 lots (3/3) positive for peanut proteins. In two other chocolate products and one snack, 2/3 lots were positive for peanuts. One cereal product had 2/4 lots positive, and all the other products had only one positive lot in total (1/1, *n* = 3; 1/2, *n* = 2; 1/3, *n* = 7; 1/4, *n* = 2; 1/5, *n* = 3). The cookie with a peanut-free claim only had 1/5 lots positive (4^th^ lot), which gave only one positive sample for the peanut-free category.

Most samples came from products manufactured in Canada (*n* = 495; 57%), with 181 samples imported from USA (21%), 119 imported from Europe (14%), and 76 (9%) imported from other countries such as China, Colombia, Mexico, etc. Of the 31 positive samples with PAL, 19 were manufactured in Canada (19/31; 61%), 6 in USA (6/31; 19%) and 6 in Europe (6/31; 19%). Positive samples with peanuts as a minor ingredient (*n* = 6) were manufactured in the USA (4/6) and in Canada (2/6). The positive sample with a peanut-free claim was manufactured in Canada.

A total of 236 samples had a private brand (manufactured by a given food company but sold under a store brand), and 635 a regular brand. Eight positive samples had a private label, and 23 a regular label, and the peanut protein concentrations varied between 0.6–9.6 ppm, and 0.6–28.1 ppm, respectively.

Statistical difference tests did not show any differences in peanut protein content between food categories, PAL statements, origin of the products and/or brand types, and because of the low number of samples (*n* < 20) of each variable, differences in the results cannot be interpreted for statistical significance.

### Hazelnut occurrence

A total of 863 samples were analyzed for hazelnuts, most bearing a PAL statement (807/863; 94%) (Table 1) and 6% (56/863) bearing a nut-free statement (Table 2). Hazelnut proteins were found in 78 samples (9%), with a range of 0.4 to 2167 ppm. All positive samples had a PAL for tree nuts and/or hazelnuts, which gives a slightly higher occurrence when considering only products with PAL (78/807; 10%). No samples with a nut-free claim were positive for hazelnut proteins.

Like peanuts, regardless of the allergen, the most popular PAL was “may contain” (*n* = 768; 89%), then “may contain traces” (*n* = 44; 5%) and other wordings (*n* = 33; 4%) to indicate the possible presence of tree nuts and/or hazelnuts. The remaining samples (*n* = 18; 2%) did not present a PAL at all for tree nuts and/or hazelnuts but presented a nut-free statement.

**Fig. 3:**
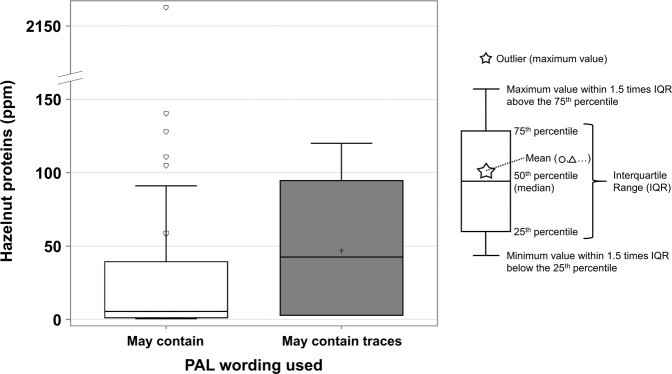
Hazelnut protein concentration by PAL statement used. Each box corresponds to a different PAL wording and has a different color-symbol combination. The “May contain” category presents five outliers, with its mean between the 75^th^ percentile and the upper whisker. There were no positive results for “May contain ingredients” or other PAL wordings in products with a PAL for tree nuts/hazelnuts.

Of the 78 samples with PAL for tree nuts and/or hazelnuts, and 71 samples presented “may contain” (0.4–2167 ppm), and seven “may contain traces” (2.8–120 ppm). The differences between the hazelnut protein concentration of both PAL statements are presented in Fig. 3. According to Fig. 3, “may contain” shows many outliers (extreme results), while 75% of its observations are below the mean and median of “may contain traces”. However, no statistical differences were found between the two PAL statements (*p* > 0.05).

**Fig. 4:**
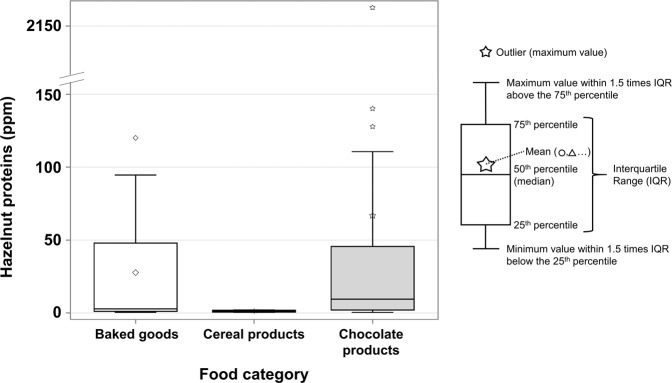
Hazelnut protein concentration by food category. Each box corresponds to a different food category and has a different color-symbol combination. The “Baked goods” category has one outlier, while “Chocolate products” has three with its mean between the 75^th^ percentile and the upper whisker. The other food categories (Baking mixes, Candies, Cookies, Savoury biscuits, Snacks) had no positive results for hazelnuts.

Like peanuts, high variations were found between food categories. As shown in Fig. 4, only the baked goods (*n* = 12; 0.5–120 ppm), cereal products (*n* = 4; 0.4–2.1 ppm), and chocolate products (*n* = 62; 0.4–2167 ppm) categories had positive samples. Thus, the hazelnut occurrence of each food category is 9% for baked goods (12/129), 4% for cereal products (4/112), and 42% for chocolate products (62/148) within products with PAL. Chocolate products is the food category that has the highest results and variations, with extreme results shown by the outliers and the mean above 75% of the observations for the baked goods category (Fig. 4).

**Fig. 5:**
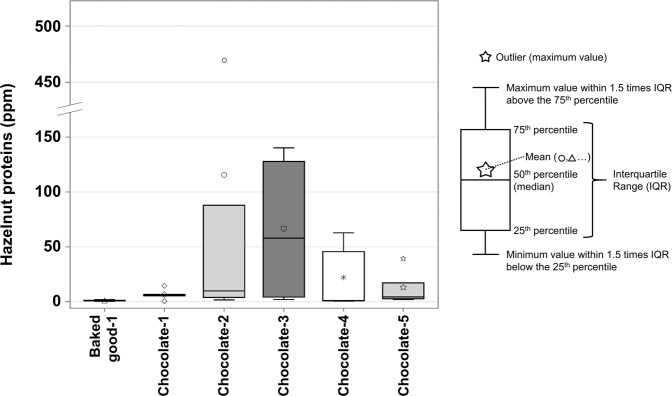
Variation in hazelnut protein concentration between five lots of six different products with 5/5 lots positive for hazelnut. Each box corresponds to a different product (chocolate or baked good) and has a different color-symbol combination. Each box (or product) displays the results of the five positive lots for hazelnuts. The product “Chocolate-2” presents one outlier. There were no other products with 5/5 lots positive for hazelnuts.

Twenty-six (26) products contained hazelnuts in one or more lots. From those 26 products, 15 (58%) contained hazelnuts in all their lots, when 2 (*n* = 2), 3 (*n* = 7) or 5 lots (*n* = 6) were analyzed. Thus, one chocolate and one baked good had 2/2 lots positive for hazelnuts, one baked good and six chocolate products contained hazelnuts in 3/3 lots, and another baked good and five other chocolate products had 5/5 lots positive. Figure 5 shows the variations between the five lots of each product who contained hazelnuts in 5/5 lots. As shown in Fig. 5, 5/6 products were chocolate samples. The mean of “Chocolate-2” (bigger circle) is outside of the box, because of the outlier present at 474 ppm. “Chocolate-3” also shows a high variation by its width, with a lower result of 2.0 ppm and a higher result of 140 ppm. Conversely, “Baked good-1” has lower amounts of hazelnuts (0.5–1. 9 ppm) and shows lesser variations between lots.

Hazelnuts were not always present in food samples. One chocolate product contained hazelnuts in 4/5 lots, one cereal product with two other chocolate products contained hazelnuts in 3/5 lots, and one baked good with another chocolate contained hazelnuts in 2/5 lots. One chocolate contained hazelnuts in 2/3 lots, in 1/2 lots for two other chocolates, and finally one chocolate and one cereal product had 1/3 lot positive for hazelnuts. In addition, the highest result of hazelnut proteins was held by a chocolate product whose 3 lots were all positive, and the protein concentrations found were respectively of 2167, 9.2, and 45.3 ppm hazelnut proteins.

When some lots were positive, the variations could also be high. One chocolate product containing hazelnuts in the first and fourth lots had hazelnut protein concentrations of 9.9 and 0.5 ppm respectively, and the remaining lots were between the LOD and the LOQ. Both products were labeled with the same PAL confirming that using a PAL can mean very different levels of contamination. Another chocolate had a smaller variation, with its second, fourth and fifth lots between 2.7 and 5.5 ppm of hazelnut proteins.

Most analyzed samples were manufactured in Canada (*n* = 413; 48%), followed by products imported from Europe (*n* = 194; 22%), USA (*n* = 186; 22%), and other countries (*n* = 70; 8%). Ten (10) positive samples were Canadian (10/78; 13%), and 68 were European (68/78; 87%). Most positive samples from Europe were chocolates (62/68; 91%). The other positive samples from Europe were one cereal product and five baked goods.

There were more samples analyzed from regular (*n* = 592) than private brands (*n* = 271). However, there were more positive samples for hazelnuts coming from private (*n* = 43; 16%) than regular brands (*n* = 35; 6%). The hazelnut protein ranges per brand type were respectively 0.5–474 ppm and 0.4–2167 ppm for private and regular brands. No statistical differences were found between the hazelnut protein concentration of the two brand types (*p* > 0.05).

### Validation

Spiking experiments using a peanut or hazelnut solution gave recoveries around 95–110%. Incurred spiking (peanut or hazelnut added in an allergen-free cookie recipe) also gave results of 98 to 105% for peanuts and 80 to 86% for hazelnuts, both in raw and baked cookies. There was no significant difference between raw and baked cookie samples for both allergens.

## Discussion

To the authors’ knowledge, no studies from other groups offered to analyze five different lots in more than 800 food samples for peanuts and hazelnuts allergens. Few of the analyzed samples with PAL did contain the allergen mentioned in the PAL statement (5% positive for peanuts and 9% for hazelnuts). While the occurrence of peanut and hazelnuts found in selected Canadian with PAL products is low, it is in accordance with most previous occurrence publications^6,8,9,11,13–15,20^. Conversely, Pele et al. (2007) found higher occurrences but focused exclusively on cookies and chocolates. In their study, higher occurrences of hazelnuts were observed in chocolates than cookies^10^, which was also our case as 42% of chocolates were positive for hazelnuts, while cookies (0%) and other categories (4–9%) had lower occurrences.

The protein ranges found for peanuts and hazelnuts with PAL were relatively low compared with milk^21,22^ but were similar to most studies^6,9,11,13–15^. The protein range found for both allergens were 0.6 to 28.1 ppm peanut proteins and 0.4 to 2167 ppm for hazelnut proteins. Considering a serving size of 40 g for any of the products analyzed, the dietary intake (or exposure) value would range between 0.02–1.12 mg and 0.02–86.7 mg for peanuts and hazelnuts, respectively. Compared to the VITAL 3.0 updated thresholds, set to offer a protection of 99% of the allergic population (0.2 mg for peanuts and 0.1 mg for hazelnuts)^18^, it is unlikely that products with smaller exposure values would trigger an objective allergic reaction (0.02 mg < VITAL doses). Regarding the highest intake values (1.12 mg for peanuts and 86.7 mg for hazelnuts), according to Houben et al. 2020, an objective reaction would be experienced by up to 4% and 24% of peanut and hazelnut-allergic individuals, respectively^23^. Thus, allergic reactions can be expected to occur for both allergens, especially hazelnuts, because of its high concentrations.

The highest hazelnut concentration was found in a chocolate bar where all three lots analyzed contained hazelnuts (2167, 9, and 45 ppm hazelnut proteins). According to these findings, and as seen in another publication from the same group^22^, heterogenous cross-contamination can occur within a same product between different industrial productions. It is likely that if some allergic consumers have a risky behavior, they may encounter foods with low and high allergen concentrations (>1000 ppm) which will likely trigger objective allergic reactions. The highest result corresponded to approximately 14,500 ppm of whole hazelnuts, equivalent to 1.45 g hazelnuts per 100 g chocolate bar (1.45% of the bar). Even if the presence of hazelnut was due to manufacturing practices and unavoidable cross-contamination, the hazelnut content should have been considered as an ingredient. As a comparison, according to the Canadian Food and Drug Regulations, emulsifying agents are considered as ingredients when added in chocolate at a level that does not exceed 1.5% of the total content (https://laws-lois.justice.gc.ca/PDF/C.R.C.,_c._870.pdf), which stands close to the 1.45% of hazelnuts in the chocolate bar. This emphasizes the need for allergen action levels (thresholds) that would help differentiate allergen cross-contamination from ingredient levels, even when the presence of the allergen is unintended.

On the other hand, 3/9 samples with peanuts listed as a minor ingredient (present as the last ingredient in the ingredients’ list) did not contain peanuts. Usually, when an allergen is present as an ingredient, it is expected to be found during testing: adding peanuts in the ingredients’ list requires the company to add a small amount during processing. This method should not be used to avoid the use of PAL if it is not going to be intentionally added in the end. One of the products was a snack using peanuts and/or soybean oil in the ingredients’ list, with peanuts listed under the “Contains” section, and had 2/3 lots positive for peanuts. The low peanut protein concentrations found (0.6 and 1.3 ppm) suggest peanut cross-contamination or the use of non-refined peanut oils (higher risk of containing peanut proteins^24^), since refined oils should not include peanut protein and therefore not pose a risk to peanut-allergic consumers^25^. In the two cereal products, 1/3 lot was negative for each product. Maybe the peanut amount added into the recipe was too small to be detected, or the matrix was not homogenous enough, and the chosen lots did not contain a detectable quantity of allergen. This is consistent with other studies the presence of peanuts as a minor ingredient where not all products tested positive. Remington et al. (2013) found that 6/16 products with peanuts as a minor ingredient were positive, and 5/6 products had both lots positive, but Robertson et al. (2013) did not find any peanut or tree nut traces in products with tree nuts (0/2) or peanuts (0/7) as minor ingredients in their samples. This suggests peanuts would not be intentionally added during food processing, or peanut and tree nut proteins could be affected during processing, thus giving negative results.

For the cookie sample bearing a peanut-free claim, a small peanut protein concentration (0.7 ppm) was found in his fourth lot (out of five). It is unlikely that this concentration would lead to an allergic reaction, since the allergic consumer would have to eat 286 g of those cookies to reach the VITAL threshold for peanuts (0.2 mg)^18^ and trigger an objective allergic reaction in 1% of the allergic population. While the occurrence is low (0.4%; 1/234), as seen in a report created by the Canadian Food Inspection Agency in 2013 (https://www.inspection.gc.ca/food-safety-for-industry/food-chemistry-and-microbiology/food-safety-testing-bulletin-and-reports/2011-2012-undeclared-allergens-in-snack-foods/eng/1430828165806/1430828166463), it remains worrying that products with allergen-free claims, deemed as safe for allergic consumers, can contain traces of allergen. The use of PAL should be considered if the food manufacturer cannot guarantee the absence of allergen in all lots.

Five lots were chosen to have a higher number than previous publications where authors used four at most^13^ to address lot-to-lot (inter-lot) variability. The results of this study show that peanut cross-contamination is heterogeneous between industrial productions (lots) and is not always present. In one baked good, 1/5 sample was positive for peanuts, which was the third lot. In a cereal product, it was the fourth lot that was positive for peanuts. It means that for these products, PAL is used correctly (cross-contamination cannot be always avoided). For the remaining products, the use of PAL could be improved. Maybe the selected lots were not the ones containing peanuts and/or hazelnuts, which brings out the importance of sampling at least 5 different lots, as this study suggests, when validating allergen presence in food products. One limitation of this sampling approach is that the intra-lot variability has not been addressed. Because tree nuts and peanuts can be present in the form of particulates (e.g., allergen residues on equipment or working surfaces), as mentioned in the newest Codex Guidelines (http://www.fao.org/fao-who-codexalimentarius/sh-proxy/en/?lnk=1&url=https%253A%252F%252Fworkspace.fao.org%252Fsites%252Fcodex%252FStandards%252FCXC%2B80-2020%252FCXC_080e.pdf), they can be distributed unevenly within the same production. Additionally, as the nature of food is mostly heterogenous, the chosen samples may not have been representative of the entire lot of that industrial production (whether the allergen was present, or not present, during analysis), explaining high variations and existence of samples containing >1000 ppm. This could have been addressed by buying multiple samples with the same lot number to have a better representativity for each lot, but then it would not have been possible to analyze as many different foods.

It was not possible to get 5 lot numbers for each food product, because by the time the study started, some products became unavailable over time (discontinued), their formulation changed (e.g., peanut were added as ingredients) or the PAL was removed or modified.

Furthermore, finding products for hazelnuts specifically, instead of tree nuts, was not always possible as the availability of such products was found to be limited. “May contain tree nuts” was present more often. If products were selected when hazelnuts were specified in the PAL statement, more positives may have been found, however, the study would have had less samples to analyze. Additionally, “may contain tree nuts” might have included other nuts such as almonds or walnuts, and the likelihood of detecting hazelnuts in these products would be lower. Thus, strong conclusions on appropriate PAL use and allergen management by food industries cannot be made.

The most common PAL used is “may contain”, as recommended by Health Canada (https://www.canada.ca/content/dam/hc-sc/migration/hc-sc/fn-an/alt_formats/pdf/label-etiquet/allergen/precaution_label-etiquette-eng.pdf). However, many prepackaged products (8% for peanuts and 11% for hazelnuts), both manufactured in Canada and in other countries, do not follow this recommendation. Statistical analysis did not show differences between the allergen concentration of the different PAL statements used, but their low number of positives (n < 20) did not allow acceptable statistical comparisons. The ranges were similar in protein concentration, which means that “may contain traces” or other PAL wordings do not correspond to a lower risk, compared with “may contain”. This confirms findings of past occurrence studies which examined different types of precautionary labeling for peanuts and tree nuts^6,10,14^.

Many samples with PAL (50%, or *n* = 414 for peanuts; 74%, or *n* = 642 for hazelnuts) had an allergen protein concentration between the LOD and the LOQ of the kits and considered as negative. Some of the results between LOD and LOQ may be positive, and food processing as well as matrix effect can be the cause of such levels not being quantified^26–28^. In addition, 183 and 87 samples (21% and 10%) were under the LOD and were considered truly negative for peanuts and hazelnuts, respectively. The LOD is usually determined by validation tests analyzing several blank matrices (without the allergen) and comparing the results. Because the LOD is determined statistically and LOQ through a known concentration (second standard of the r-Biopharm kit), declaring positive results based on the LOQ is more reliable^28^. ELISA methods’ sensitivity could be improved to enable detection of small concentrations of analytes, as small concentrations of allergens can lead to allergic reactions. In addition, when a result falls between LOD and LOQ, it is generally advised that a kit from another company should be used to confirm the presence/absence of allergen, especially if an allergen-free claim is present.

High variations between protein concentrations were shown as the standard deviation value was higher than the mean for peanuts in chocolate products, cookies, and snacks, and hazelnuts in chocolate products and baked goods. This is explained by very high and very low allergen protein concentrations found in this study. As an example, the highest and lowest results for the presence of hazelnuts in chocolate products were also the lowest and highest results found in this study.

For both allergens, there were no positive results observed for baking mixes, candies or savoury biscuits, and the most contaminated matrix was chocolate products. The occurrence of peanuts and hazelnuts was higher in the chocolate products category, especially for hazelnuts, since 62/78 positive samples were European chocolates. Milk is still the allergen with the highest occurrence in dark chocolate, and chocolate (in general) is still the most problematic food in regards to allergen cross-contamination^9,21,22^. Indeed, chocolate is a very difficult to clean matrix since wet cleaning is not practiced because of microbiological (notably possible contamination with *Salmonella spp*.) and quality issues^29^. Dry cleaning is a common practice in chocolate manufacturing, but because it cleans partially, allergen cross-contamination is less avoidable than wet cleaning^29^.

All 62 positive chocolate products for hazelnuts were imported from Europe, which could be explained by the common use of hazelnuts in Europe in chocolates and confectionary products. To draw a comparison between Canadian and European products, more chocolate products manufactured in Canada would be needed, but the sample number would be decreased as most chocolate products available on the market were more likely to be manufactured in Europe.

The low number of positive samples for peanuts (*n* = 38) did not allow any robust statistical difference tests to be considered. Only one statistical difference test was done between brand types on hazelnut protein concentration (for *n*_positives_ > 20), but no differences were observed (*p* > 0.05). There was a higher proportion of positive samples within private brands (products sold under store brands) than regular brands, although no differences were found between their hazelnut protein concentration (*p* > 0.05). This suggests that the same care is applied for all products regardless of the brand.

The r-Biopharm peanut kit was chosen because it is part of the Performance Tested Methods^SM^ of the AOAC (https://members.aoac.org/AOAC/PTM_Validated_Methods.aspx), meaning the kit satisfied the Method Performance criteria associated with this category of validation by AOAC International for detection and quantitation of peanuts. The hazelnut kit was chosen from the same test kit provider to ensure homogeneity of the results, even if it was not identified as a Performance Tested Method^SM^ by the AOAC. However, spiking validations were performed as part of this study to ensure homogeneity in the experimental protocol for both kits. Incurred spiking was performed because most products are processed foods, and cross-contamination is more likely to occur during incorporation of raw ingredients into the recipe. Furthermore, incurred spiking was also recommended by Abbott et al. (2010), because spiking using allergen solutions tends to overestimate recovery results. Recovery results obtained were around 100%, fulfilling the requirements of the allergen method validation guidance developed by the AOAC food allergen community (i.e. acceptable recoveries between 50 and 150%)^30^. Thus, the occurrence results obtained are likely to be close to the real allergen concentration found inside analyzed products, rather than over or underestimated.

The validation approach adopted has many limitations in its design. Foremost, peanut and hazelnut standards used for spiking were not made of recognized reference material (e.g., NIST peanut butter) like r-Biopharm used to validate their commercial kits. In addition, the only food matrix tested was cookies for both peanut and hazelnut, and this study analyzed other products that may have different matrix effect, impacting the recovery. Other authors also reported high recoveries for peanuts in dark chocolate with the r-Biopharm peanut kit^27^. Such data is not available for other foods like baked goods or cereal products. Ideally, food industries should test their own food matrices within their own production conditions, as they might have different recoveries than cookies and chocolate. However, food manufacturers might not have the necessary means nor the equipment to do these validations and increase unnecessary risks of additional allergen contamination in subsequent productions. To avoid such risks, positive matrices (already containing the allergens as ingredients) sampled from industrial productions or spiked matrices in a kitchen or laboratory-like setting (for smaller allergen quantities) could be used for validation purposes.

Even if it seems that food processing such as roasting increases peanuts’ allergenicity^31,32^, and decreases allergenicity of hazelnuts when roasted or exposed to high temperatures^33,34^, according to the validation results, it is likely that even after processing, peanuts and hazelnuts could still be detected at satisfying recoveries (did not under or overestimate results drastically, which would otherwise question the veracity of the results obtained in this study).

Despite limitations due to intra-lot variability, there is a very low occurrence of samples containing the allergens in the PAL statement collected in the province of Quebec, as 95% and 90% of the samples with PAL analyzed for peanuts and hazelnuts respectively did not contain their allergen mentioned in the PAL statement and would have been safe to eat by peanut and/or hazelnut-allergic consumers. Manufacturing practices need to be improved to lead food industries to a better use of PAL (based on risk assessments and efforts to minimize cross-contamination) and avoid the use of PAL as a means to make blanket statements that are meant to limit liability. Regulatory instances might work with food manufacturers, clinicians, and consumer associations to elaborate additional guidelines and reference doses for PAL use towards the better use of PAL. Regarding the use of allergen-free claims, one sample out of 234 with a peanut-free claim contained peanuts, which is certainly low but not acceptable as these products are sought after and selected by the allergic population, because considered to safer food alternatives. Since chocolate products whose labels had a PAL were the most contaminated matrices for both allergens, they should be considered as worst-case scenarios to elaborate allergen action levels with risk assessment for all food categories, and avoided by allergic consumers, to prevent all unnecessary objective reactions.

## Methods

### Sample plan

Based on past publications from cross sectional studies and official monitoring plans from several jurisdictions^6,8–15,20,35,36^ including the CFIA food recall database due to peanuts and hazelnuts presence as allergens from 1997 to 2017 (https://inspection.gc.ca/food-recall-warnings-and-allergy-alerts/eng/1351519587174/1351519588221?ay=0%2C0&fr=0%2C0&fc=0%2C0&fd=0%2C0&ft=2%2C2), eight food categories were determined as high-risk foods inducing most recalls. These food categories include baked goods, baking mixes (e.g., cake or muffin mixes), candies, chocolate, cookies, ready-to-eat meals, savoury biscuits, and snacks. Baking mixes and Savoury biscuits were meant for peanuts only, but if a PAL for tree nuts was present on the label of a product from these food categories, it would be analyzed for hazelnuts. Ready-to-eat meals were not selected for the study as they were often associated to labeling mistakes instead of cross-contamination issues in the CFIA database. Even if they did not create many recalls in Canada, cereal products were added to the food categories because they were often analyzed and cited in the literature as a high risk food^6,8,11–15,20,35,37^.

The authors attempted to get 30 products with PAL for each food category, and up to 5 different lot numbers (i.e., from 5 different industrial productions) for each product to assess lot-to-lot variability. Depending on availability, a goal of 150 samples for each allergen per category (1200 samples per allergen) for the entire study, collected between May 2018 and February 2020.

### Sample acquisition

Food products for peanuts (*n* = 280) and hazelnuts (*n* = 286) were purchased in grocery stores and small retail stores in several locations in Quebec City, Quebec, Canada. The principal selection criterion was that the product needed to have a PAL for peanuts and/or tree nuts. Some products with broader PAL statements previously analyzed for milk and/or eggs in a previous occurrence study from the same authors^22^ were analyzed for peanuts and hazelnuts if those products had a PAL for peanuts, hazelnuts and/or tree nuts. Products with allergen-free claims (peanut-free and/or nut-free) were also investigated to confirm the veracity of the label and absence of allergen. Finally, three lots of three products (one snack and two cereal products) with peanuts as a minor ingredient (last ingredient listed on label) were analyzed to confirm their presence.

Product information was kept in a database, and included the name, brand, retailer who sold the product, date of purchase, allergens present, lot numbers and best before dates as proposed by the Food Safety Authority of Ireland for occurrence/survey studies (http://www.fsai.ie/resources_publications/allergen_labelling_2011.html.html).

### Sample preparation

After purchase, samples were grinded using Grindomix GM200 industrial grinders (Retsch, Haan, Germany) to process samples and result in a fine powder to be used in the analysis.

Foodstuffs with a high fat content (e.g., nut trail mixes), chocolate, chocolate-containing products or cream-filled foods (e.g., cookies with a cream or a fruit filling) were placed in the fridge for at least an hour prior to grinding to prevent melting (chocolate) and/or formation of a non-homogenous paste. In addition, candies (especially gums) were placed in the freezer overnight prior to grinding to also avoid the formation of a paste and result in a fine powder. Fresh products with a low shelf life like baked goods were kept in the freezer until analysis to prevent their deterioration (formation of mold).

### ELISA analysis

Samples were analyzed in duplicates using two ELISA sandwich kits from r-Biopharm, RIDASCREEN®FAST Peanut and RIDASCREEN®FAST Hazelnut (r-Biopharm, Darmstadt, Germany). The allergens were extracted according to the method provided with the kit. Results were analyzed using the RIDASOFT software from r-Biopharm. Because results are given in ppm (mg/kg) peanuts and ppm hazelnuts, conversion factors provided by the company (0.222 and 0.15 for peanuts and hazelnuts, respectively) were used to transform results to ppm proteins instead, as recommended by other authors^11^. All results above the limit of quantification (LOQ) of the kit of each allergen (0.555 ppm for peanuts and 0.375 ppm for hazelnuts) are considered positive. All results below, even above the limit of detection (LOD) were considered without detectable allergens, or negative results (0.03 ppm for peanuts and hazelnuts).

### Validation of ELISA kits

Prior to analyzing samples for this study, two spiking validations were performed. The first spiking was done using a peanut butter solution (natural peanut butter made of roasted peanuts) or a solution of grinded raw hazelnuts to spike peanut-free and hazelnut-free matrices (cookies), and recoveries (allergen proteins recovered during analysis/allergen proteins added inside the food matrix) were evaluated. Allergen spiking solutions were prepared accordingly with the instructions provided by r-Biopharm (product information leaflets, available on demand).

Another in-house validation of incurred spiking (adding 1000 ppm of the allergen in an allergen-free cookie recipe, bake the cookies at 180 °C, then evaluate the recovery) was also performed for both peanuts and hazelnuts. Five raw and five cooked samples were analyzed in duplicates for each allergen, with a blank (only reagents) and a negative control (raw ingredients without the added allergen). In all spiking validations, milk powder was added as recommended by r-Biopharm to avoid false positives or higher recoveries for peanuts and hazelnuts.

### Statistical analysis

Results were analyzed using SAS Studio University Edition, v.3.8 (Basic Edition) (SAS Institute Inc., North Carolina, USA). The peanut and hazelnut protein concentrations were compared between food categories, PAL type, country of origin, and brand type (private/store brand vs. regular brand), using PROC FREQ. PROC SGPLOT was used to design figures, and PROC MEANS and PROC UNIVARIATE were used to sort descriptive statistics and define normality of data.

Preliminary tests showed peanut and hazelnut protein concentrations were not following normal distributions. Thus, procedure PROC NPAR1WAY (nonparametric tests for non-normal data) was used to determine if statistical differences existed between the different variables (food category, PAL, origin, brand type, allergen) of this study. Wilcoxon Scores, the Kruskal-Wallis test, and the Dwass, Steel, Critchlow-Fligner (DSCF) Method for Pairwise Two-Sided Multiple Comparison Analysis were used for this procedure.

## Data Availability

Raw data is publicly available on the PARERA platform website (https://parera.ulaval.ca/news-and-events/news/article/news/mise-a-disposition-de-donnees-doccurence-dallergenes-presents-dans-les-produits-alimentaires-avec-un/).
